# An fMRI Study of Word Reading and Colour Recognition in Different Quadrant Fields

**DOI:** 10.2174/1874440000802010056

**Published:** 2008-08-12

**Authors:** Tadashi Ino, Ryusuke Nakai, Takashi Azuma, Kazuki Tokumoto, Kiyohide Usami, Toru Kimura

**Affiliations:** 1 Department of Neurology, Rakuwakai-Otowa Hospital, Otowachinjicho 2 Yamashina-ku, Kyoto 607-8062, Japan; 2 Research Center for Nano Medical Engineering, Institute for Frontier Medical Sciences, Kyoto University, Shogoin, Sakyo-ku, Kyoto 606-8507, Japan

## Abstract

This fMRI study analyzed activations for processing of word and colour, which were presented in each of the four quadrants, to investigate anatomical segregation between colour and orientation processing and also to examine the effect of visual stimulus position on brain activations. Main effect of visual category was found in the bilateral extrastriate cortices extending to the left visual word form area (word > colour) and small area of the right ventrolateral prefrontal cortex (colour > word). ROI analysis showed that there was a tendency that V4α, not V4/8, showed a greater response to colours than to words. Main effect of visual fields was found in early visual areas, which showed greater responses to the left than to the right field stimuli and also to the lower than to the upper field stimuli. No significant interactions between visual category and visual fields were found.

## INTRODUCTION

Form and colour are major attributes of the visual stimuli. Perception of form in primates is hierarchically organized from simple edge detection by orientation-selective neurons and spatial frequency-selective neurons to entire figure recognition by the ventral visual pathway [1-3]. In Macaque, it has been suggested that the inferior temporal cortex (IT) is a region critical for object recognition [4, 5]. Recent neuroimaging studies in humans showed that the lateral occipital complex (LOC) in the lateral and ventral regions of the occipital lobe is activated when processing visual stimuli of objects [6-9], suggesting that human LOC may be analogous to Macaque IT. It was postulated that different categories of visual stimuli are processed in the different areas of the upstream of the ventral visual pathway. Those regions specialized for perception and recognition of certain visual categories such as faces, images depicting places, images of bodies, or words are referred to as fusiform face area (FFA) [10, 11], parahippocampal place area (PPA) [12, 13], extrastriate body area (EBA) [14], or visual word form area (VWFA) [15, 16], respectively.

Regarding colour perception, it has been suggested that in monkeys wavelength-selective neurons are populated in V4 [17-19]. Subsequently, neuroimaging studies in humans have identified a region that is specifically involved in colour processing in the fusiform gyrus or collateral sulcus [20-25]. Although this colour centre in the human brain area was named V4 by Zeki and his colleagues (almost identical area was named V8 [22] or VO [26]), human V4 should not be regarded as homologous with monkey V4 due to anatomical reason [27] (but see [28, 29]). Moreover, the claim that monkey V4 is a colour centre was questioned by later studies [30-32].

It was reported that colour-sensitive neuron that code orientation as well as colour (oriented colour-sensitive neurons) are found in monkey V1 [33-35] and in human visual areas including V4/8 [36]. Previous studies, which were performed to localize colour centre, used rather complex chromatic figure such as Mondrian pattern and contrasted it with identical figure without colour. This contrast will extract activities of oriented colour-sensitive neurons in addition to non-oriented colour-sensitive neurons to colour. On the other hand, previous studies regarding receptive field and function of monkey V1 showed that perception of the colour of a surface depends on neural activities evoked by the border of the surface rather than its interior and a uniform colour figure activates cortical cells representing the borders, but few if any of those representing the interior [37, 38]. However, a recent study showed that about 20 % of neurons in monkey V1 and V2 are highly responsive to the interior of uniform colour surface [39]. Furthermore it was reported that human colour centre complex (V4 and V4α) were activated by a uniform colour stimulus [40]. Therefore, in the present study, we used uniform colour stimuli that will activate selectively non-oriented colour-sensitive neurons in order to estimate the extent to which cortical regions that process colour are segregated from those that process orientation. If activities of the non-oriented, colour-sensitive neurons are dominant over the activities of the orientation-sensitive, non-colour-sensitive neurons, we expect that this colour centre will show a greater response to uniform colour stimuli than to colourless figure stimuli. On the other hand, if the activities of the orientation-sensitive, non-colour-sensitive neurons are dominant over activities of the non-oriented, colour-sensitive neurons, we expect that this colour centre will show a greater response to achromatic figure than to uniform colour. In order to minimize difference of demands on the higher order brain function between colour task and figure task, one kanji character which denotes a colour was used as a figure stimulus, and subjects had to perform the same behaviour to colour and figure stimuli. Single kanji letter is strongly associated with semantics and therefore it can be regarded as a word. Consequently, the VWFA which is postulated to be involved in processing the prelexical representation of visual word form, albeit the concept of which has been challenged [41-43], is expected to be identified in the comparison between kanji and colour task. Verifying this is the second purpose of the present study.

The visual stimuli were presented in the four quadrants for the following reasons. Human psychological and electrophysiological studies indicated lower visual field advantage in complex visual processing [44-46]. Recent MEG studies also showed that activities of the early visual cortex are stronger for lower than upper visual stimuli of black-and-white checkerboards [47], grating pattern [48] and faces [49]. Right visual field (RVF) advantage over left visual field (LVF) in visual word processing was also reported. For example, lexical decision to words, semantic decision to words, and naming to words were faster in the RVF than in the LVF [50,51]. The neural basis of lower and left visual field advantage has not yet been examined systematically using functional neuroimaging methods. Therefore, we compared the brain activation between the left visual field stimuli and the right visual field stimuli and also between the lower visual field stimuli and the upper visual field stimuli. Since human ability to recognize spatial and colour stimuli declines with increasing eccentricity, relatively large images of word and colour were placed in the parafoveal region in order to enable subjects to recognize them with ease while maintaining central fixation. Although eye movements were not monitored during fMRI study, all participants were trained to fixate at the central mark outside the scanner, and we confirmed that they could perform the task without difficulty.

## METHODS

### Participants

Eighteen volunteers (10 males and 8 females), aged 21-31 year (26.4 ± 3.2, mean ± SD), participated in this study. All subjects were right-handed and their scores for the Japanese version of the Edinburgh handedness inventory [52] were all above +80. All subjects were free of any psychiatric or neurological illness and had normal or corrected-to-normal vision and normal colour vision. The experimental procedure was approved by the Medical Ethics Committee at Otowa Hospital, and all participants gave written informed consent.

### Procedure

**Fig. (1):**
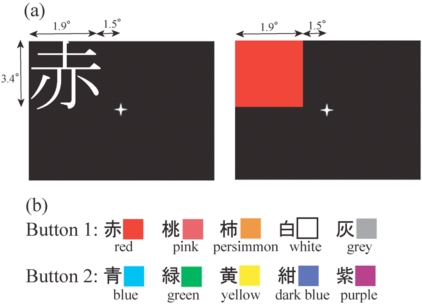
**(a):** The representative image presentation in the left upper quadrant. **(b):** The rule of kanji/colour to button correspondence.

Echo-planar images (TR = 2 sec, TE = 50 msec, flip angle = 90(, FOV = 200 mm, matrix = 64 x 64, slice thickness = 4 mm, gap = 1 mm, 25 axial slices) and a three-dimensional T1 weighted image for anatomical normalization (TR = 11.6 msec, TE = 4.9 msec, flip angle = 8(, FOV = 220 x 220 mm, matrix = 256 x 256, slice thickness = 1 mm) were obtained using a 1.5-T Siemens Sonata. The fMRI study consisted of three conditions: word reading, colour recognition and rest. The subject was instructed to fix their eyes on the mark presented in the centre of the screen throughout all conditions. During word reading condition, colour names written in a single kanji character were presented at one of the four quadrants. Subjects had to press one button using the index finger if the kanji letter was one of the five letters which denotes either red end of the spectrum (red, pink, persimmon), white, or grey, and to press another button using the middle finger if the kanji letter was one of five letters which denotes other colours, namely, blue, green, yellow, dark blue, and purple (Fig. **1**). During colour recognition condition, a coloured square was presented at one of the four quadrants, and subjects had to press one switch by index finger when the colour was either red end of the spectrum, white, or grey, and to press another switch by middle finger when the colour was some other. During rest, subjects were required to simply fix their eyes on the central mark. Before the examination in the scanner, subjects were trained to master the kanji/colour to button correspondence. The stimulus images were projected onto a transparent screen, using a personal computer with custom written software and a projector, which the subject viewed through a mirror attached to a head coil. Images of a word or coloured square were presented for 800 msec with a blank interval of 800 msec. Each epoch of word reading (W) and colour recognition (C) in either of four quadrant (LUp (left upper), LLo (left lower), RUp (right upper), and RLo (right lower)) lasted 16 sec, during which 10 images of word or colour were presented, and they were interleaved with rest (R), which also lasted 16 sec. In order to counterbalance fMRI adaptation, namely, fMRI signal reduction due to repeated presentation of the same visual stimuli [8, 53] across four quadrants, nine subjects did in the sequence R/W-LUp/C-LUp/R/C-RUp/W-RUp/R/W-RLo/C-RLo/R/C-LLo/W-LLo/R/W-LLo/C-LLo/R/C-RLo/W-RLo/R/W-RUp/C-RUp/R/C-LUp/W-LUp/R x 2 and the other nine subjects did in reverse order, namely, R/W-LLo/C-LLo/R/C-RLo/W-RLo/R/W-RUp/C-RUp/R/C-LUp/W-LUp/R/W-LUp/C-LUp/R/C-RUp/W-RUp/R/W-RLo/C-RLo/R/C-LLo/W-LLo/R x 2. By sequencing this way, summation of the stimulation order was equal in each quadrant, and the order of word and colour in each quadrant was counterbalanced. The total scanning session lasted 804 sec including a 10 sec dummy scan to allow for stability in magnetization. There were four epochs of word and colour task for each quadrant, and one of four fonts types and one of four different colour shades were provided to each epoch for each quadrant.

### Data Analysis

The fMRI data was analyzed using SPM2. After realignment, normalization, and smoothing with 6-mm FWHM Gaussian filter, condition-related response was modelled by a boxcar convolved with a canonical hemodynamic response function. High pass filter with 1/128 Hz cut off frequency was used to remove low-frequency signal drifts. Motion parameters defined by the realignment procedure were added to the model as six regressors of no interest. At this stage, the parameter estimates for each of eight conditions (i.e. word or colour x four quadrants presentation) were computed. The data was subjected to a repeated-measures ANOVA including a correction for non-sphericity. We then looked for the main effects of visual category (colour *vs. *word) and visual fields (left *vs. *right and lower *vs. *upper) and their interaction. Activated areas were regarded as significant if more than 20 voxels exceeded P < 0.01, corrected for multiple comparisons using false discovery rate unless otherwise stated.

## RESULTS

### Behavioural Results

Behavioural data of two subjects were lost due to technical error. For remaining 16 subjects, reaction times and accuracy rate of word and colour condition in each quadrant are shown in Table **1**. The data was subjected to a 2-factor repeated measures ANOVA with task (colour *vs. *word) and image position (LLo, LUp, RLo, RUp). For reaction times, there was a main effect of colour *vs. *word (F (1, 15) = 21.5, P < 0.0001). Post hock t – test with Bonferroni correction showed that reaction times for word condition were significantly longer than those for colour condition (P < 0.0001). There was no significant difference in response times between image position (F (3, 13) = 0.491, P = 0.694), and task x image position interaction was not significant (F (3, 13) = 1.121, P = 0.376). There was no significant difference in response accuracy between word and colour (F (1, 15) = 1.116, P = 0.308), between image position (F (3, 13) = 1.365, P = 0.297), or their interaction (F (3, 13) = 0.016, P = 0.997).

### fMRI Results

#### Main Effect of Visual Category

**Fig. (2):**
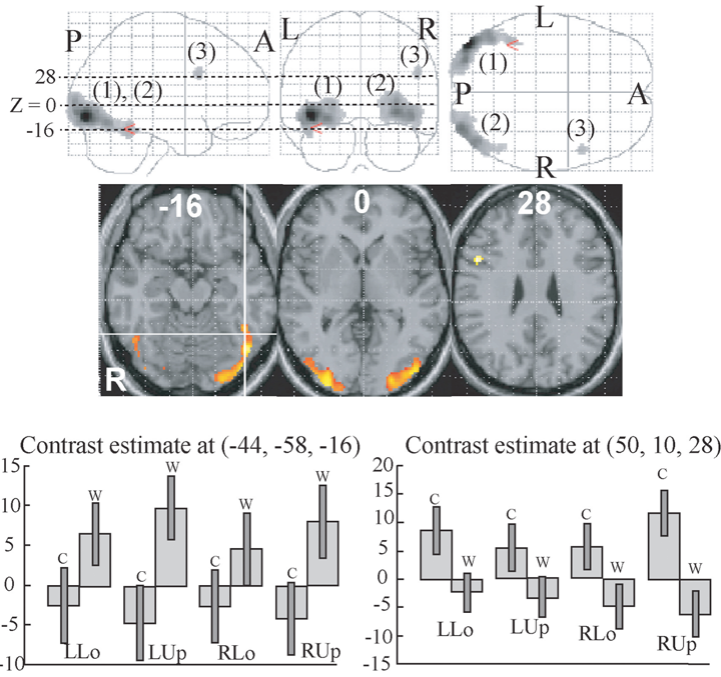
Regions whose activities were significantly different between colour and word task (P < 0.01, corrected for multiple comparisons using false discovery rate, k > 20 voxels). They are displayed on a glass brain (upper) and on the axial images of the MNI template (middle).Figures in glass brain and the axial images refer to the axial coordinates of MNI space. The red arrowhead in the upper and the intersection oflines in the middle correspond to the centre of the VWFA defined by meta-analysis [54]. Two large clusters (1, 2) showed greater activationfor words than for colours and one small cluster (3) showed greater activation for colours than words. Contrast effect of the centre of theVWFA defined by meta-analysis (lower left) and that of the activation peak of the cluster (3) (lower right). Bars represent the limit of the 90% confidence interval around the mean.

Two clusters whose activations were greater for words compared to colours were identified in the bilateral extrastriate visual areas (Fig. **2** (1), (2), Table **2**). The left cluster extended to the centre of the VWFA, defined by the meta-analysis of 16 neuroimaging studies during word reading (x = -44, y = -58, z = -15 in MNI coordinates [54]). As shown in Fig. (**2**), the activation in this voxel was greater for words than colours in all quadrant presentation. One small cluster whose activation was greater for colours than for words was identified in the right ventrolateral prefrontal cortex (Fig. (**2**) (3), Table **2**). As shown in Fig. (**2**), the activation of the peak of this cluster was greater for colours than words in all quadrant presentation.

#### ROI Analysis of the Activation of V4/8 and V4α

**Fig. (3):**
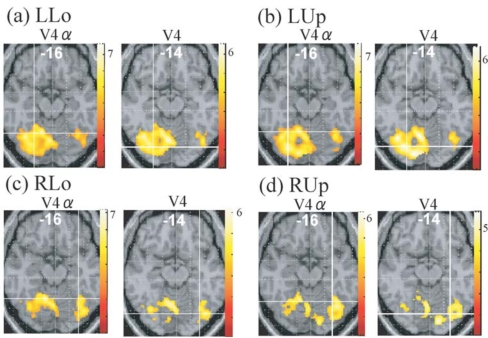
Regions whose activities were greater for colour task than for rest in each quadrant presentation (P <0.01, corrected for multiplecomparisons using false discovery rate, k > 20 voxels), displayed on the axial images where putative V4 or V4 exists. Intersection of linescorresponds to the averaged coordinates obtained in the individual subject analysis (cf. Fig. **4**). LUp, left upper; LLo, left lower; RUp, rightupper; RLo, right lower.

Although no cluster whose activation was greater for colours than for words was found in the visual cortex even when the level of significance was set more liberal to p < 0.05, k > 20, uncorrected for multiple comparisons, large visual areas including V4/8 and V4α which were reported to be located in the postero-ventral area in the fusiform gyrus or collateral sulcus [23, 24] showed significant activation (P < 0.01, k > 20, corrected for multiple comparisons using false discovery rate) for colour task compared to rest in all four quadrant presentation (Fig. **3**). This finding suggests that even though non-oriented, colour-sensitive neurons were localized in the colour centre, they were colocalized with orientation-sensitive, non-colour-sensitive neurons. To estimate the degree of colocalization of colour and orientation processing in the colour centre, we then compared the activation of the V4/8 and V4α between colour and word condition by ROI analysis. We defined putative colour centres (V4/8 and V4α) in the fusiform gyrus or collateral sulcus that showed significant activation (P < 0.001, uncorrected) for colour task compared to rest in each quadrant presentation for each subject. Adjusted signal change was calculated by sampling voxels within a 5-mm-diameter volume around the peak activation for each quadrant presentation for each subject, and we averaged activation within the last 10 s of each epoch to exclude delayed BOLD activity from the preceding epoch. Adjusted signal change of the same voxels for word task for each quadrant presentation for each subject was also calculated. The data was subjected to paired t-test (two-tailed). As shown in Fig. (**4**), there was a tendency that V4α was activated more strongly for colours than for words in every quadrant presentation, although V4 did not show such a trend.

#### Main Effect of Visual Field

The right V1 and adjacent V2 showed a greater response to the left than to the right visual stimuli and the left V1 and adjacent V2 showed a greater response to the right than to the left visual stimuli (Fig. **5**, Table **3**). The cluster size of the former was larger than that of the latter (166 *vs. *71) and peak z-score of the former was higher than that of the latter (5.9 *vs. *5.3) (Table **3**). Two clusters ((1, 2) in Fig. **5**) of V1 and adjacent V2 showed greater responses to the lower than to the upper visual stimuli and one cluster (3) in Fig. **5**) of V1 showed a greater response to the upper than to the lower visual stimuli. The cluster size of the former was larger than that of the latter (274 *vs. *32) and peak z-score of the former was higher than that of the latter (6.6 vs.5.1) (Table **3**).

#### Visual Category by Visual Field Interaction

No voxel exceeded the threshold in the analysis of the visual category (colour *vs. *word) by visual fields (left *vs. *right and lower *vs. *upper) interaction.

## DISCUSSION

**Fig. (4):**
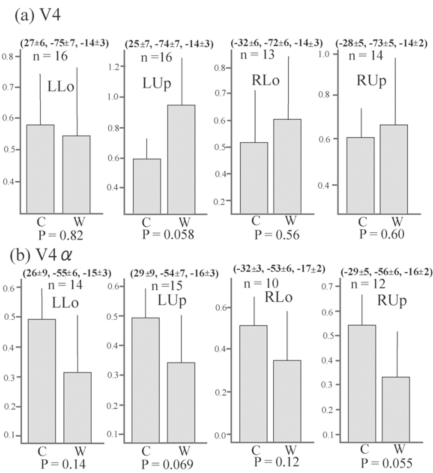
Average adjusted signal change of the putative V4 and V4α . The MNI coordinates (mean ± SD) and number of sampled subjects isalso shown. The number of subjects was not constant since only subjects who showed significant activation for each contrast were selected.There is a tendency that the signal changes of putative V4α , but not of putative V4/8, were greater for colour task than for word task in allquadrant presentation. Bars represent the limit of the 95% confidence interval around the mean.

Although oriented colour-sensitive neurons will respond to both colour and word stimuli, activation of them will be decreased due to habituation since every word had white colour and every colour stimuli had identical figure. Therefore, the comparison between activation for word task and that for colour task will reflect mainly a difference between responses of non-oriented, colour-sensitive neurons to colours and those of orientation-sensitive, non-colour-sensitive neurons to words. Although visual areas including V4/8 and V4α were extensively activated for colour task compared to rest (Fig. **3**), no visual areas showed a significantly greater activation for colour task than for word task in the whole brain analysis. ROI analysis showed that there was a tendency that V4α showed a greater response to colours than to words in every quadrant presentation whereas V4/8 did not show such a trend (Fig. **4**). These results suggest that numerous orientation-sensitive, non-colour-sensitive neurons are colocalized with colour-sensitive neurons in V4/8 whereas the proportion of the former neurons to the latter neurons is relatively low in V4α. Although the latter suggestion should be interpreted with caution since the area of the colour image was much larger than that of the word image, and therefore the comparison of colour *vs. *word condition will be overestimated, it can be said that the activity of orientation-sensitive, non-colour-sensitive neurons relative to the activity of non-oriented, colour-sensitive neurons is lower in V4α than in V4/8. This may indicate that V4α is hierarchically higher than V4/8 for colour processing and appears to conform to the previous report showing that V4/8 is retinotopically organized while V4α dose not have a retinotopic organization [24].

Two large clusters, which showed a greater response to words than to colours, were identified in the bilateral ventral extrastriate visual cortices (Fig. **2** (1), (2), Table **2**). Although mapping of the visual areas were not performed in the present study, based on the previous mapping study of visual areas which provided visual maps calibrated in the Talairach coordinates and anatomical properties [55-58] those clusters extended from V2 to the anterior-ventral direction involving V3, V4, and LOC. On the left side, they further extended to the posterior occipitotemporal sulcus, which was reported to have enhanced efficiency for processing visual word forms [59], and involved the centre of the VWFA [54]. This finding confirmed our a priori prediction that one kanji letter will be processed in the brain as a word. The posterior-to-anterior flow of information from bilateral extrastriate cortices though bilateral LOC to the left occipitotemporal sulcus corresponds to transition from pattern analysis to visual language function. This schema is consistent with a recently proposed model (the local combination detector model [60]) and with a recent finding that successively more anterior region of the occipitotemporal cortex was activated by visual stimuli with an increasing structural similarity to real word [61]. However, we do not think that VWFA is specialized for word reading in a strong sense. A recent fMRI study showed that activation of the VWFA was greater for object naming than word reading [62]. A more recent PET study also showed that rCBF in the VWFA was higher to object than to word when task demand for object is high, and it was proposed that activation of the VWFA reflects the integration of shape elements into elaborate shape description corresponding to whole objects or words [63]. When we confine our attention to neuronal activities associated with word reading, VWFA will be a place to which visual information converges and from which it diverges to language areas. However, a significant number of neurons, whose function is related to object recognition and not to word reading, will be located in the VWFA. Nevertheless, the VWFA appears to be a bottleneck for word reading and not for object recognition or naming, and that is why disturbance of object naming was relatively mild in patients who developed severe alexia following injury of the VWFA.

One small cluster in the right ventrolateral prefrontal cortex showed a greater response to colours than to words (Fig. **2** (3), Table **2b**). This region may be related to certain type of working memory processing for non-verbal materials, albeit precise significance of this finding is unclear. Although reaction time was significantly longer for word than colour task, no region in the fronto-parietal cortex including attention networks was activated greater for word than for colour task, suggesting that word-specific cognitive load was confined to relatively low order visual analysis.

**Fig. (5):**
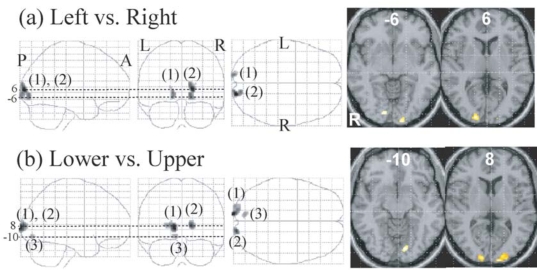
Regions whose activations were different between the left and right presentation **(a)** and between the lower and upper presentation **(b)** (P <0.01, corrected for multiple comparisons using false discovery rate, k > 20 voxels). They are displayed on a glass brain (left) and onthe axial images of the MNI template (right). Figures refer to the axial coordinates of MNI space. **(a)** The left V1/2 (1) showed a greater responseto the right than to the left field stimuli and the right V1 (2) showed a greater response to the left than to the right stimuli. **(b)** Twoclusters of V1/2 (1, 2) showed a greater response to the lower than to the upper stimuli and one cluster of V1 (3) showed a greater responseto the upper than to the lower stimuli.

Main effect of visual field (left *vs. *right and lower *vs. *upper) was found in V1 and V2 adjacent to V1. One reason for the absence of the effect in other visual areas by a random effect whole-brain analysis is that later visual areas have a large receptive visual field for which there is very significant variability in retinotopic areas across subjects. In the comparison between brain activations for the left field visual stimuli and those for the right field visual stimuli, the right V1 and V2, which were activated more strongly for the left stimuli than for the right stimuli, were more noticeable than the left V1 and V2, which were activated more strongly for the right stimuli than for left stimuli (Fig. **5**, Table **3**), suggesting that V1 and V2 were activated more robustly by the visual stimuli presented in the left hemifield than those presented in the right hemifield. A recent study showed that attention-related brain regions such as intraparietal sulcus, frontal eye field, dorsolateral prefrontal cortex and thalamus were bilaterally activated more strongly by the left visual stimuli than by the right stimuli [64]. However, this left visual field superiority of an attention network was not observed in the present study. This discrepancy is probably due to task difference. Subjects focused their attention on the stimuli displayed on the right or left of the fixation point in our study, whereas subjects were instructed to report colour change of the fixation point to direct attention away from the stimuli presented to the left or right of the fixation point in the previous study [64].

In the comparison between brain activations for the lower field visual stimuli and those for the upper field visual stimuli, V1/V2 which were more activated for the lower stimuli than for the upper stimuli were more noticeable than V1/V2 which were more activated for the upper stimuli than for the lower stimuli (Fig. **5b**, Table **3c,d**), suggesting that early visual areas were more robustly activated to the visual stimuli presented in the lower field than to those presented in the upper field, and in line with the previous MEG study showing significantly stronger activation of the V1/V2 cortex to lower than to upper field visual stimuli such as checkerboard [47], grating pattern [48] and faces [49].

There was no region where significant interaction between visual category (colour *vs. *word) and visual fields (lower *vs. *upper and left *vs. *right) was found, suggesting that the main effect of word and colour task is not significantly different between left and right presentation and also between lower and upper presentation and that the preference of V1/2 activity for the left over the right hemifield and for the lower over the upper visual field is not significantly different between word and colour task.

Behaviourally, right than left [50, 51] and lower than upper visual field advantages [44-46] were reported. However, neither reaction time nor accuracy rate was significantly different between visual fields in the present study, probably due to ceiling effect. It has been reported that efficiency of word recognition in the right and left visual fields were similar for words with less than 3 letters, although words with longer letters presented in the right visual field were recognized more efficiently than those presented in the left visual field [65, 66]. The lower visual field, which has been reported to be advantage for behavioural performance in the previous studies, was also advantage for V1 activation in the present fMRI study. On the other hand, the right visual field, which has been reported to be advantage for behavioural performance, was disadvantage for V1 activation in the present fMRI study. Left visual field superiority for V1 activation may suggest that V1 activity is controlled by visuospatial attention network, which is lateralized to the right side of the brain. Alternatively, activity of the right V1 to the left visual stimuli should be strong to overcome the handicap that information from it must go to the opposite hemisphere to be processed in the language areas.

## CONCLUSION

The current fMRI study analyzed activations for processing of a word representing a colour and a coloured square, both of which were presented in each of the four quadrants, to investigate anatomical segregation between colour and orientation processing and also to examine the effect of visual stimulus position on brain activations. The main effect of word > colour was associated with extensive bilateral extrastriate activation including the left visual word form area. The main effect of colour > word was associated with activation of a region in the right ventrolateral prefrontal cortex. Although visual areas including V4/8 and V4α were broadly activated during colour task compared to rest, no visual region showed a significantly greater activation for colours than for words in the whole brain analysis. Subsequent ROI-based analyses revealed a tendency for greater activation of V4α during colour task compared with word task whereas no such tendency was apparent in V4/8. These finding suggest that a certain level of segregation of colour and orientation processing may be present in V4α, and therefore V4α is hierarchically higher than V4/8. Main effect of visual fields (left *vs. *right and lower *vs. *upper) was found in early visual areas, which showed greater responses to the left than to the right and also to the lower than to the upper field stimuli. No significant interaction between visual category (colour *vs.*word) and visual fields (lower *vs. *upper and left *vs. *right) was observed.

## Figures and Tables

**Table 1.** Behavioural Data

	Reaction Times (msec)	Accuracy Rate (%)
**Word-LLo**	674 ± 89	97.7 ± 3.2
**Colour-LLo**	637 ± 80	97.2 ± 3.2
**Word-LUp**	672 ± 88	98.3 ± 3.3
**Colour-LUp**	648 ± 83	97.8 ± 3.4
**Word-RLo**	668 ± 92	97.8 ± 2.7
**Colour-RLo**	650 ± 96	97.2 ± 3.3
**Word-RUp**	667 ± 83	97.3 ± 3.6
**Colour-RUp**	641 ± 83	97.2 ± 4.3

**Table:** (a) Word > Colour

Anatomical Area	BA	Side	x	y	z	Z Score
Inferior occipital gyrus	19	L	-42	-86	-8	Inf
	19	R	42	-80	-6	7.2
	18	L	-28	-88	-10	7.6
	18	R	32	-92	-6	7.8
Middle occipital gyrus	19	L	-48	-68	-12	7
	19	R	46	-80	-10	7.1
	18	L	-24	-94	-2	6.1
	18	R	30	-96	2	6.8
Fusiform gyrus	37	L	-44	-50	-22	5.5
	37	R	50	-60	-16	4.5
Inferior temporal gyrus	37	L	-54	-58	-12	3.7

**Table:** (b) Colour > Word

Anatomical Area	BA	Side	x	y	z	Z Score
Ventrolateral PFC	44	R	50	10	26	5.1

**Table:** (a) Left > Right

Anatomical Area	BA	Side	x	y	z	Z Score	Voxels
Cuneus	17	R	18	-94	6	5.9	166

**Table:** (b) Right > Left

Anatomical Area	BA	Side	x	y	z	Z Score	Voxels
Cuneus	17	L	-14	-96	0	5.3	71

**Table:** (c) Lower > Upper

Anatomical Area	BA	Side	x	y	z	Z Score	Voxels
Cuneus	18	L	-12	-100	6	6.6	188
	17	R	16	-96	6	6.1	86

**(d):** (d) Upper > Lower

Anatomical Area	BA	Side	x	y	z	Z Score	Voxels
Cuneus	17	L	-10	-82	-10	5.1	32

## References

[1] Ungerleider LG, Mishkin Ingel DJ, Ingel  DJ, Mansfield RJW, Goodale MS M (1982). Two cortical visual systems The analysis of visual behaviour. The analysis of visual behaviour.

[2] Van Essen DC, Maunsell JHR (1983). Hierarchical organization and functional stream in the visual cortex. Trends Neurosci.

[3] Van Essen DC, Anderson CH, Fellman DJ (1992). Information processing in the primate visual system an integrated systems perspective. Science.

[4] Logothetis NK, Shinberg DL (1996). Visual object recognition. Annu Rev Neurosci.

[5] Tanaka K (1996). Inferotemporal cortex and object vision. Annu Rev Neurosci.

[6] Malach R, Reppas JB, Benson RR (1995). Object-related activity revealed by functional magnetic resonance imaging in human occipital cortex. Proc Natl Acad Sci USA.

[7] Grill-Spector K, Kushnir T, Edelman S, Itzchak Y, Malach R (1998). Cue-invariant activation in object-related areas of the human occipital lobe. Neuron.

[8] Grill-Spector K, Kushnir T, Edelman S, Avidan G, Itzchak Y, Malach R (1999). Differential processing of objects under various viewing conditions in the human lateral occipital complex. Neuron.

[9] Kourtzi Z, Kanwisher N (2000). Cortical regions involved in perceiving object shape. J Neurosci.

[10] Kanwisher N, McDermott J, Chun MM (1997). The fusiform face area: a module in human extrastriate cortex specialized for face perception. J Neurosci.

[11] Kanwisher N, Stanley D, Harris A (1999). The fusiform face area is selective for faces not animals. Neuroreport.

[12] Epstein RA, Kanwisher N (1998). Cortical representation of the local visual environment. Nature.

[13] Epstein RA, Harris A, Stanley D, Kanwisher N (1999). The parahippocampal place area: recognition, navigation, or encoding?. Neuron.

[14] Downing PE, Jlang Y, Shuman M, Kanwisher N (2001). A cortical area selective to visual processing of the human body. Science.

[15] Cohen L, Dehaene S, Naccache L (2000). The visual word form area: spatial and temporal characterization of an initial stage of reading in normal subjects and posterior split-brain patients. Brain.

[16] Cohen L, Lehericy S, Chochon F, Lemer C, Rivard S, Dehaene S (2002). Language-specific tuning of visual cortex? Functional properties of the visual word form area. Brain.

[17] Zeki S (1973). Colour coding in rhesus monkey prestriate cortex. Brain Res.

[18] Zeki S (1977). Colour coding in the superior temporal sulcus of rhesus monkey visual cortex. Proc R Soc London B.

[19] Zeki S (1978). Uniformity and diversity of structure and function in rhesus monkey prestriate visual cortex. J Physiol (Lond.).

[20] Sakai K, Watanabe E, Onodera Y (1995). Functional mapping of the human colour centre with echo-planar magnetic resonance imaging. Proc R Soc Lond B.

[21] McKeefry DJ, Zeki S (1997). The position and topography of the human colour centre as revealed by functional magnetic resonance imaging. Brain.

[22] Hadjikhani N, Liu AK, Dale AM, Cavanagh P, Tootell RBH (1998). Retinotopy and colour selectivity in human visual cortical area V8. Nat Neurosci.

[23] Zeki S, Bartels A (1999). The clinical and functional measurement of cortical (in)activity in the visual brain, with special reference to the two subdivisions (V4 and V4?) of the human colour centre. Philos Trans R Soc Lond B.

[24] Bartels A, Zeki S (2000). The architecture of the colour centre in the human visual brain: new results and a review. Eur J Neurosci.

[25] Wade AR, Brewer AA, Rieger JW, Wandell BA (2002). Functional measurements of human ventral occipital cortex retinotopy and colour. Philos Trans R Soc Lond B.

[26] Wandell BA (1999). Computational neuroimaging of human visual cortex. Annu Rev Neurosci.

[27] Tootell RBH, Hadjikhani N ( 2001). Where is dorsal 'v4' in human visual cortex? retinotopic, topographic and functional evidence. Cereb Cortex.

[28] Brewer AA, Liu J, Wade AR, Wandell BA (2005). Visual field maps and stimulus selectivity in human ventral occipital cortex. Nat Neurosci.

[29] Wandell BA, Brewer AA, Dougherty RF (2005). Visual field map cluster in human cortex. Philols Trans R Soc Lond B.

[30] Fisher B, Boch R, Bach M (1981). Stimulus versus eye movements: comparison of neural activity in the striate and prelunate visual cortex (A17 and A19) of trained rhesus monkey. Exp Brain Res.

[31] Schein SJ, Marrocco RT, de Monasterio FM (1982). Is there a high concentration of colour-selective cells in area V4 of monkey visual cortex?. Neurophysiology.

[32] Schein SJ, Desimone R (1990). Spectral properties of V4 neurons in the macaque. J Neurosci.

[33] Leventhal AG, Thompson KG, Liu D, Zhou Y, Ault SJ (1995). Concomitant sensitivity to orientation, direction, and colour of cells in layers 2, 3 and 4 of monkey striate cortex. J Neurosci.

[34] Michael CR (1978). Colour-sensitive complex cells in monkey striate cortex. J Neurophysiol.

[35] Michael CR (1979). Colour-sensitive hypercomplex cells in monkey striate cortex. J Neurophysiol.

[36] Engel SA (2005). Adaptation of oriented and unoriented colour-selective neurons in human visual areas. Neuron.

[37] Hubel DH, Wiesel TN (1968). Receptive fields and functional architecture of monkey striate cortex. J Physiol.

[38] Hubel DH (1988). Eye, Brain, and Vision. Scientific American Library.

[39] Friedman HS, Zhou H, von der Heydt R (2003). The coding of uniform colour figures in monkey visual cortex. J Physiol (Lond.).

[40] Morita T, Kochiyama T, Okada T, Yonekura Y, Matusmura M, Sadato N (2004). The neural substrates of conscious colour perception demonstrated using fMRI. Neuroimage.

[41] Price CJ, Devlin JT (2003). The myth of the visual word form area. Neuroimage.

[42] Price CJ, Winterburn D, Giraud AL, Moore CJ, Noppeney U (2003). Cortical localization of the visual and auditory word form areas: a reconsideration of the evidence. Brain Lang.

[43] McCrory EJ, Mechelli A, Frith U, Price J (2005). More than words: a common neural basis for reading and naming deficits in developmental dyslexia?. Brain.

[44] Skrandies W, Ottoson  D (1987). The upper and lower visual field of man Electrophysiological and functional differences. In Progress in sensory physiology Springer. Berlin Heidelberg.

[45] Previc FH (1990). Functional specialization in the lower and upper visual fields in humans its ecological origins and neurophysiological implications. Behav Brain Sci.

[46] Rubin N, Nakayama K, Shapley R (1996). Enhanced perception of illusory contours in the lower versus upper visual hemifields. Science.

[47] Portin K, Vanni S, Virsu V, Hari R (1999). Stronger occipital cortical activation to lower than upper visual field stimuli-neuromagnetic recordings. Exp Brain Res.

[48] Tzelepi A, Ioannides AA, Poghosyan V (2001). Early (N70m) neuromagnetic signal topography and striate and extrastriate generators following pattern onset quadrant stimulation. Neuroimage.

[49] Liu L, Ioannides A (2006). Spatiotemporal dynamics and connectivity pattern differences between centrally and peripherally presented faces. Neuroimage.

[50] Ellis EW (1988). Modes of visual word recognition in the left and right cerebral hemispheres. Brain Lang.

[51] Bub D, Lewine J (1988). Different modes of word recognition in the left and right visual fields. Brain Lang.

[52] Oldfield RC (1971). The assessment and analysis of handedness the Edinburgh inventory. Neuropsychology.

[53] Martin A, Lalonde FM, Wiggs CL, Weisberg J, Ungerleider LG, Haxby JV (1995). Repeated presentation of objects reduces activity in ventral occipitotemporal corlex: a fMRl study of repetition priming. Soc Neurosci Abst.

[54] Jobard G, Crivello F, Tzourio-Mazoyer N (2003). Evaluation of the dual route theory of reading a metaanalysis of 35 neuroimaging studies. Neuroimage.

[55] Shipp S, Watson JDG, Frackowiak RSJ, Zeki S (1995). Retinotopic maps in human prestriate visual cortex the demarcation of area V2 and V3. Neuroimage.

[56] DeYoe EA, Carman GJ, Bandettini P (1996). Mapping striate and extrastriate visual areas in human cerebral cortex. Proc Natl Acad Sci USA.

[57] Hasnain MK, Fox PT, Woldorff MG (2001). Structure-function spatial covariance in the human visual cortex. Cereb Cortex.

[58] Rottschy C, Eickhoff SB, Schleicher A, Mohlberg H, Zilles K, Amunts K (2007). Ventral visual cortex in humans cytoarchitectonic mapping of two extrastriate areas. Hum Brain Mapp.

[59] Ben-Shachar M, Dougherty RF, Deutsch GK, Wandell BA (2007). Differential sensitivity to words and shapes in ventral occipito-temporal cortex. Cereb Cortex.

[60] Dehaene S, Cohen L, Sigman S, Vinckier F (2005). The neural code for written words: a proposal. Trends Cogn Sci.

[61] Vinckier F, Dehaene S, Jobert A, Dubus JP, Sigman M, Cohen L (2007). Hierarchical coding of letter strings in the ventral stream: dissecting the inner organization of the visual word-form system. Neuron.

[62] Price CJ, Mccorory E, Noppeney U, Mechelli A, Moore CJ (2006). How reading differs from object naming at the neural level. Neuroimage.

[63] Starrfelt R, Gerlach C (2007). The Visual What For Area: words and pictures in the left fusiform gyrus. Neuroimage.

[64] Siman-Tov T, Mendelsohn A, Schonberg T (2007). Bihemispheric leftward bias in a visuospatial attention-related network. J Neurosci.

[65] Young AW, Ellis AW (1985). Different methods of lexical access for words presented in the left and right visual fields. Brain Lang.

[66] Ellis EW (2004). Length, formats, neighbours, hemispheres, and the processing of words presented laterally or fixation. Brain Lang.

